# Horiba Micros ES 60 Blood Cell Analyzer in Blood Donor Eligibility: A Validation Study

**DOI:** 10.3390/diagnostics12112586

**Published:** 2022-10-25

**Authors:** Silvia Tillati, Ilaria Pati, Michela Delle Donne, Alessandra Meneghel, Donatella Londero, Vincenzo De Angelis

**Affiliations:** 1 Unit of Medical Statistics, Department of Clinical and Experimental Medicine, University of Pisa, 56121 Pisa, Italy; 2 National Blood Centre, Italian National Institute of Health, 00161 Rome, Italy; 3 Transfusion Medicine Department, Udine University Hospital, 33100 Udine, Italy

**Keywords:** blood donation, blood component quality, hematology analyzer, quality control, donor eligibility

## Abstract

Background: Eligibility criteria for blood donation require hemoglobin levels of ≥12.5 g/dL for women and ≥13.5 g/dL for men, and a platelet count of ≥180 × 109/L. Screening methods before donation should ensure high accuracy, precision, and ease in operation. We assessed the performance, precision, and repeatability of the Horiba Micros ES 60 (Horiba) compared to the Beckman Coulter DXH 800. Methods: Performance was compared by testing samples for each of the 11 devices across 6 sites in the Transfusion Service of Friuli Venezia Giulia Region, Italy. We measured precision by calculating the coefficient of variation (CV), concordance with ρ-Pearson’s correlation coefficient, and accuracy with F-tests. The intra-assay agreement was examined in the 11 devices, and repeatability was performed by using CV and the Kruskal–Wallis test. Results: The precision of Horiba was acceptable. Overall, ρ-Pearson’s coefficients indicated a strong correlation and positive relationship between all variables. The Bland–Altman plots showed that most of the differences lay within the limits of agreement. All CV were below the reference threshold for all the parameters. Finally, the Kruskal–Wallis test reported non-significant statistical differences for all parameters, except platelet count (*p* < 0.000). Conclusions: Horiba is adequate for routine pre-donation screening. The intra-assay agreement further demonstrates the accuracy of the device.

## 1. Introduction

The donation interval and maximum frequency of donation may have long-term consequences on the development of iron deficiency (ID) and also anemia in donors [1]. Eligibility criteria for whole blood donation require minimal acceptable hemoglobin (HBG) levels of 12.5 g/dL for women and 13.5 g/dL for men for protecting donor’s health and guaranteeing quality standards of blood components [2,3]. According to the current transfusion legislation, in Italy the detection of HBG, with the performance of the complete blood count, must be performed before each donation [3].

Another important variable for ensuring the quality of blood components is the platelet (PLT) count, since the efficiency of PLT collection with cell separators is related to the initial value of the donor peripheral PLT count (and possibly with donor hematocrit). The eligibility criteria for apheresis donation require a PLT before donation of not less than 180 × 10^9^/L [2]. Furthermore, for the apheresis donation of two units of PLT concentrate, the pre-donation PLT count cannot be less than 200 × 10^9^/L [3]. Peripheral PLT concentration determination performed immediately before the apheresis procedure is of great help for a more accurate and precise prediction of final PLT yield. These criteria are taken into account by apheresis machines in calculating the volume of blood to be processed for a defined target of PLT harvest. Consequently, the PLT count must be performed before each apheretic procedure, according to the current Italian legislation [3].

Several methods of screening are used to test HBG in donors before donation, mainly requiring a finger-stick, and also non-invasive ones have been proposed; although, they are not consistently regarded as having proven effectiveness [4]. There is no general consensus on a pre-donation PLT count methodology.

The desirable features of a device intended for screening include high accuracy, precision, rapidity, and ease of operation and these goals must be achieved in the blood centers, as well as in mobile units [4]. A standard protocol should be in place in blood collection centers to check the quality of results [2]; a validation protocol must be followed, and validation should include comparison to a full blood count measured on a venous sample.

The first aim of this study is to assess the performance on blood count parameters’ measurements required by current legislation, as well as on HBG and PLT, of the ABX Micros ES 60 automated hematology analyzer compared to the Beckman Coulter DXH 800. The secondary aim is to assess the precision of the influence of various sources on operating conditions: repeatability, reproducibility, and evaluating test results concerning the intra-assay agreement of replicates.

## 2. Materials and Methods

### 2.1. Blood Sample Collection

This study was performed on paired and non-paired capillary and/or venous blood samples (the latter anticoagulated with K2EDTA) taken by experienced nurses from regular donors of the Transfusion Medicine Department of Udine University Hospital and from outpatients undergoing chronic transfusion therapy with RBC concentrates for chronic anemia. The study has been performed through the flow cytometric analysis of 40 venous and finger-prick blood samples for each of the 11 Horiba Micros ES 60, manufactured by Horiba ABX SAS Company (Montpellier, FR, USA), vs. Beckman Coulter DXH 800 devices, manufactured by American medical company Beckman Coulter (Miami, FL, USA), across 6 different sites (Palmanova, Latisana, Cividale, San Daniele, Tolmezzo, and Udine (with six collection sites)) in the Regional Health Authority of Friuli Venezia Giulia, Italy.

### 2.2. Characteristics of the Hematology Analyzers

The tool used in this study, installed at the Department of Transfusion Medicine of Area Vasta of Udine, is a quantitative multi-parameter, automated hematology analyzer for in vitro diagnostic use in ABX Micros ES 60 of the company Horiba ABX SAS, interfaced bidirectionally with the Emonet information system. The analyzer is used to identify and enumerate the following parameters: WBC, RBC, HGB, HCT, MCV, MCH, MCHC, RDW, PLT, MPV, LYM%, LYM#, MON%, MON#, GRA%, GRA#, in K2EDTA, and K3EDTA anticoagulated venous whole blood samples of the adult patient population. Whole blood micro-sampling is 10 μL.

For comparison, the samples tested with this instrument were processed on the fully automated blood analyzer DXH 800 of the company Beckman Coulter DXH 800, interfaced in a bidirectional way with the information system Dnlab, supplied at the Laboratory of Analysis of the University Hospital of Udine. The analyzer DXH 800 provided 28 hematologic parameters, including a complete blood count (CBC) and differential count, corrected white blood cell (WBC) count, nucleated red blood cell (NRBC) count, and 4 reticulocyte (RET) parameters. Aspiration volumes were equal to 165 µL.

Both instruments were subjected to internal quality controls daily before the start of the analytical activity.

### 2.3. Measured and Calculated Parameters

We measured and calculated the following parameters with their reference units: hemoglobin (HGB, g/dL), platelets (PLT, 109/L), red blood cells (RBC, 106/mm^3^), white blood cells (WBC, 106/mm^3^), hematocrit (HCT, %), and mean corpuscular volume (MCV, fl). The analytical methods of each parameter are defined by the manufacturer in the instrument specifications. In detail, the minimum required blood sample volume was 50 μL, whereas the minimum analyzer sample volume was 10 μL; dilution ratios relative to WBC and RBC/PLT were approximately 1/260 and 1/15,000, respectively. Measurements and computation for each parameter were the following: (1) impedance change for parameters WBC, RBC, and PLT; (2) spectrophotometry for HBG; (3) numeric integration for HCT; and (4) computation from stored data that were directly measured for MCV [5].

### 2.4. Data Analysis

A validation plan (VP) for the screening test has been designed and the diagnostic method was validated in terms of assay precision, repeatability, and diagnostic accuracy in capillary vs. venous blood [6].

#### 2.4.1. Comparison between Horiba Micros ES 60 and Beckman Coulter DXH 800

The VP investigated the correlation between the data obtained from the analysis of 40 capillary blood samples taken by digitopuntura from the donor population vs. the venous blood samples of the donor population taken by venipuncture, during their periodic monitoring. The same operator took both the capillary and venous samples from the same donor, while different operators took samples from different donors.

To ascertain whether the Horiba Micros ES 60 analyzer had an acceptable performance relative to Beckman Coulter DXH 800, we measured the precision, the concordance, and the accuracy [7]. Samples were also transported to the central lab for the measurement of the relevant blood parameters on the Beckman Coulter DXH 800. We calculated: (1) precision by using the coefficient of variation (CV); (2) correlation by calculating the linear regression (ρ-Pearson’s coefficient) between values assessed by Horiba Micros ES 60 and the measured values obtained with Beckman Coulter DXH 800; and (3) accuracy assessed by measuring, from the blood samples collected from each individual using the two different instruments, means, standard errors, and variance of all relevant parameters. A Fisher’s paired sample F-test was performed to evaluate for each parameter any difference in variance. *p* values less than 0.01 were deemed as statistically significant. The Bland–Altman plot [8], or difference plot, is a graphical method to compare two measurements techniques and to plot the difference scores of two measurements against the mean for each subject.

#### 2.4.2. Intra-Assay Agreement Horiba Micros ES 60

Assay precision refers to the influence of various sources (i.e., operators, instruments, location) on operating conditions. Therefore, intra-series accuracy was ensured by 10 consecutive repetitions of 3 fresh venous blood samples for 3 consecutive days, taken by venipuncture from the donor population. The same operator carried out the 10 consecutive repetitions, while different operators carried out the repetitions on different days.

The within-run precision (repeatability) of the Horiba Micros ES 60 analyzer was examined by performing 10 replicate analyses of three different venous samples in three consecutive days with a total of nine venous samples for each of the 11 devices.

The following minimum standard CV thresholds were provided by the manufacturer in the instrument specifications: HBG < 1.5%, PLT < 5%, RBC < 2%. WBC < 2.5%, HCT < 2%, and MCV < 1.5% were considered [5].

A stepwise approach was carried out to evaluate the dependence of the CV values with respect to the three days of detection. First, we assessed the dependence between CV values and different days of detection for each device. Second, we analyzed all data and compared them between the individual devices. Third, with the normal tests of Shapiro–Walk and Kolmogorov–Smirnov, we analyzed the normality of the distributions of the six parameters. Fourth, we used nonparametric tests, in particular the Kruskal–Wallis test to verify the existence of a difference in median between the overall CV values and the individual devices [9].

## 3. Results

### 3.1. Comparison between Horiba Micros ES 60 and Beckman Coulter DXH 800

**Figure 1:**
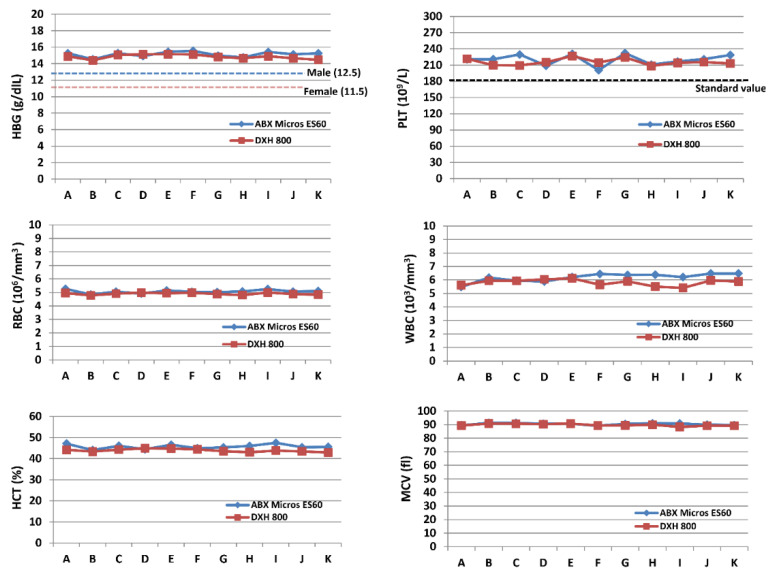
Graphical comparison between Horiba Micros ES 60 and Beckman Coulter DXH 800. Mean values for all the devices with reference value. The progressive letters on the ordinate axis refer to the 11 tested devices.

The precision CV values reported between Horiba Micros ES 60 and Beckman Coulter DXH 800 appeared perfectly overlapped for all the parameters. ρ-Pearson’s coefficients ranged between 82% and 94%, thus indicating a strong correlation and positive relationship between the variables. The F-test indicated statistically significant differences in the variances only for RBC and HCT (Table 1). The means’ values reported between Horiba Micros ES 60 and Beckman Coulter DXH 800 values appeared to be substantially overlapped for all parameters (Figure 1).

**Figure 2:**
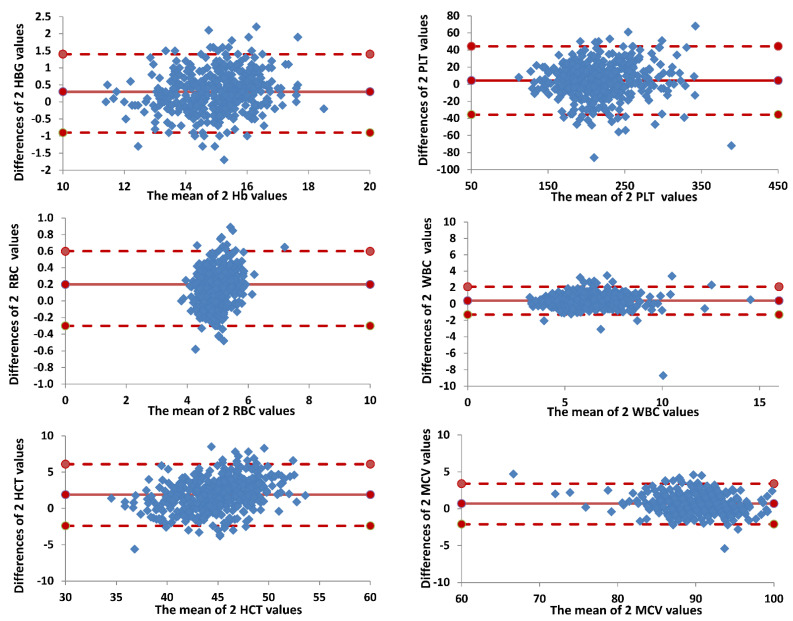
Agreement between Horiba Micros ES 60 and Beckman Coulter DXH 800. Bland–Altman plot of agreement between two values (overall data). Horizontal lines are drawn at the mean difference, and at the limits of agreement, which are defined as the mean difference plus and minus 1.96 times the standard deviation of the differences.

The Bland–Altman plots confirmed the previous results since most of the differences (between values assessed by Horiba Micros ES 60 and the measured values obtained with Beckman Coulter DXH 800) plotted against the averages of the two measurements lay within the limits of agreement, defined as the mean difference plus and minus 1.96 times the standard deviation of the differences (Figure 2).

For technical-organizational reasons it was not possible to carry out the analysis on a larger sample, and this could represent a limitation of our study.

### 3.2. Intra-Assay Agreement Horiba Micros ES 60

**Figure 3:**
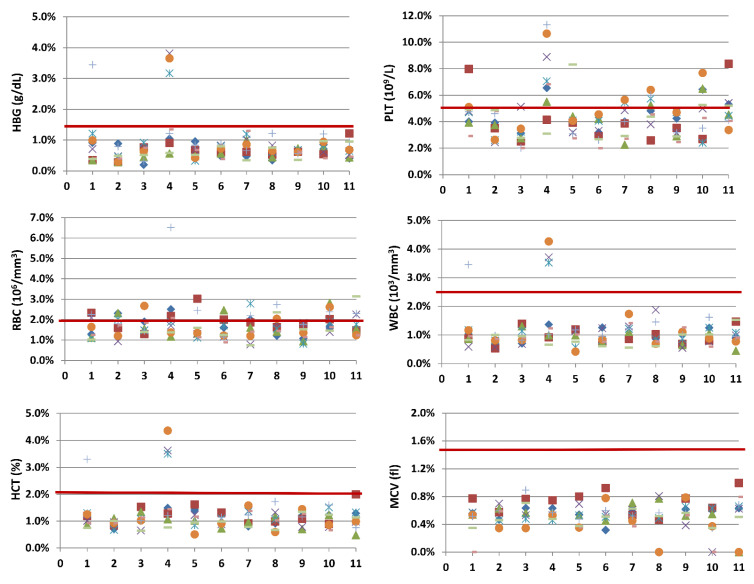
Graphical intra-assay agreement Horiba Micros ES 60.CV for all the devices’ sample with threshold (CV reference value). The progressive numbers on the ordinate axis refer to the 11 tested devices.

All the CV calculated for each day of the surveys presented values below the reference threshold for all the parameters. We confirmed that there was no statistically significant dependence between the CV values and the different days of detection. Indeed, the coefficients of correlation for each parameter under examination indicated no statistically significant dependence between the values of CV and the different sites. Therefore, we were able to describe for each parameter the CV values with the main statistical indices. Results from the Kruskal–Wallis test indicated a non-significant statistical difference in the median for all the parameters, except for PLT (*p* < 0.0001) (Table 2), as also shown in Figure 3.

## 4. Discussion

Iron deficiency, with or without anemia, is an unwanted effect inconstantly affecting repeat blood donors, and sometimes requiring iron supplementation [10,11,12]. Among the objectives of the RISE (REDS-II Donor Iron Status Evaluation) study included the evaluation of the effects of blood donation intensity on iron and HBG status. It showed that in the United States, among 2425 men and women belonging to geographically and demographically diverse populations, accepted for red blood cell blood donation, 66% of the frequent women donors and 49% of the frequent men donors were iron deficient. The study concluded that the prevalence of iron deficiency and HBG deferrals would likely be reduced by increasing the inter-donation interval or by implementing iron supplementation [12].

The HBG of a blood donor drops by 1–1.5 g/dL after a single whole blood donation, while in very frequent plasmapheresis (i.e., up to 100 donations per year) or plateletpheresis donors, iron depletion may occur due to the small but significant amount of red cells remaining in the circuits of the machines, the cell lysis, and the samples drawn for tests [4,13]; hence, an appropriate HBG pre-donation test may mitigate the possibilities of causing the blood donor to become anemic. At the same time, pre-donation HBG screening is also aimed at assuring the quality of blood components, which has implications for the health of the recipient.

Pre-donation HBG in peripheral blood is often considered an indirect measurement of iron status, due to the obvious fact that an iron-deficient ineffective erythropoiesis leads to anemia; nevertheless, studies show that HBG is not a good indicator of iron stores [14]. At the stage where anemia occurs, iron deficiency is almost invariably severe, and donors must be deferred from blood donation, and their reintroduction into the active donor population is at risk. Several methods of screening are used to test HBG in blood donors before donation: gravimetric method by finger-prick, spectrophotometric devices, and full blood count by capillary samples. The latter seems to be the mostly used technology.

Due to different pre-analytical as well as analytical factors, capillary blood is said to be an estimate rather than a measure of the venous HBG value. Furthermore, finger-stick values vary with respect to venous by donor characteristics (age, race, sex, and general health, smoking, and physical activity), but also based on the blood collection centre [4]. In most iron-depleted females and in some iron-depleted males, their finger-stick samples overestimate when the venous HBG value is near the cutoff [15].

Some studies report CMV as a useful screening tool to detect iron deficiency and hemoglobinopathy [16]. Therefore, it is advisable to consider hemocytometric parameters as a whole to evaluate anemia in blood donors.

Another parameter to evaluate, for the quality and safety of donation, is the PLT count for the plateletpheresis donation. For this reason, it is important that plateletpheresis is performed on the actual PLT count and not on historical data. The collection of PLT in apheresis has reached a good level of safety; although a significant decrease in complete blood count is observed in donors, the risk of thrombocytopenia or anemia appears to be contained [13,14,15,16,17]. Furthermore, the frequency of donation does not appear to have a negative effect on the coagulation of regular donors [18].

As with HBG, the change in PLT count is not only related to the frequency of donation, but also to the characteristics of donors: gender and age seem to influence the values. In particular, female donors have a higher PLT count than males, and this parameter decreases with increasing age [19]; iron deficiency results in a higher PLT count than donors with adequate iron stores. External factors, such as seasonality, appear to be involved in a lower PLT count, recorded mainly in the warm months [20]. However, the HBG and PLT values must be evaluated with blood count for donor eligibility.

Since, as mentioned above, the PLT and HBG values can be influenced by numerous factors, it is important that devices intended for screening have high accuracy, precision, rapidity, and ease of operation.

A comparative analysis between the Horiba Micros ES 60 automated hematology analyzer and Beckman Coulter DXH 800 showed concordant results between the two devices in terms of precision, concordance, and accuracy. A statistical analysis highlighted a strong correlation and positive relationship among the variables analyzed, and the Fisher test did not show statistically significant evidence in the variances of the analyzed parameters, with the exception of RBC and HCT. Since this variability does not affect all parameters, it may be related to the sampling phase or to the operator’s handling of the sample. Furthermore, the intra-assay agreement shows statistically significant differences in medians only for PLT values (Table 2, Figure 3). Since these differences are not observed for the other parameters, they are not attributable to the individual device, but it is conceivable that they are mainly linked to the sample characteristics. Both the type of donor and external factors may have determined the wide variability recorded. These results, therefore, do not show differences in the performance of the two analyzed devices, and the Horiba Micros ES 60 analyzer is suitable for use in routine activities.

Certainly, the use of suitable devices has a significant impact on the quality and safety of blood transfusion as it helps to identify the donor at risk, avoiding a further depletion of iron and PLT reserves. In addition, the use of the Horiba Micros ES 60 analyzer would allow to screen, in a practical, fast, and reliable way, the donors, avoiding the collection of units not suitable for use and therefore destined to be discarded.

## 5. Conclusions

The Horiba Micros ES 60 analyzer is adequate for routine pre-donation screening investigations, for the parameters measured in the study. The intra-assay agreement further demonstrates the accuracy of the device. The significant differences emerged on the PLT count are not attributable to the precision limits of the devices used, but presumably to a sample variability.

## Figures and Tables

**Table 1:** Comparison between Horiba Micros ES 60 and Beckman Coulter DXH 800. Mean, standard deviation (SD), coefficient of variation (CV), ρ-Pearson’s coefficient, and *p*-value F-test.

	Horiba Micros ES60			Beckman Coulter DXH 800			ρ-Pearson’s Coefficient	*p*-Value F-Test (*)
	Mean	SD	CV	Mean	SD	CV		
HGB (g/dL)	15.14	1.23	0.08	14.84	1.13	0.08	0.88	0.041
PLT (10^9^/L)	220.23	44.37	0.20	215.86	42.97	0.20	0.89	0.251
RBC (10^6^/mm^3^)	5.07	0.46	0.09	4.90	0.41	0.08	0.88	0.004
WBC (10^3^/mm^3^)	6.19	1.58	0.26	5.81	1.55	0.27	0.84	0.356
HCT (%)	45.72	3.82	0.08	43.86	3.09	0.07	0.82	0.000
MCV (fl)	90.38	3.93	0.04	89.72	4.20	0.05	0.94	0.082

(*) *p*-value < 0.01 was considered as statistically significant.

**Table 2:** Intra-assay agreement Horiba Micros ES 60. Descriptive statistics and test of CV value overall (mean, standard deviation (SD), Kruskal–Wallis test).

	HGB (g/dL)	PLT (10^9^/L)	RBC (10^6^/mm^3^)	WBC (10^3^/mm^3^)	HCT (%)	MCV (fl)
threshold	<1.5%	<5%	<2%	<2.5%	<2%	<1.5%
mean	0.8%	4.4%	1.1%	1.7%	1.2%	0.6%
lower limit (95% CIs)	0.7%	4.0%	1.0%	1.6%	1.1%	0.5%
upper limit (95% CIs)	0.9%	4.7%	1.2%	1.9%	1.3%	0.6%
median	0.7%	4.1%	1.0%	1.6%	1.1%	0.5%
SD	0.6%	1.8%	0.6%	0.7%	0.6%	0.4%
min	0.2%	2.0%	0.4%	0.7%	0.5%	0.0%
max	3.8%	11.3%	4.3%	6.5%	4.4%	3.5%
IQ	0.4%	2.0%	0.4%	0.7%	0.4%	0.1%
Kruskal–Wallis test (*)	20.46 (0.025)	32.95 (0.000)	13.80 (0.182)	12.27 (0.269)	14.27 (0.161)	10.63 (0.387)
Median test (*)	18.95 (0.041)	20.58 (0.024)	13.26 (0.210)	7.65 (0.663)	18.94 (0.041)	8.77 (0.554)

(*) *p*-value < 0.01 was considered as statistically significant.

## Data Availability

The data supporting the study results can be found at Transfusion Medicine Department, Udine University Hospital, 33100 Udine, Italy.
